# Quantitative Evaluation of Kidney and Gallbladder Stones by Texture Analysis Using Gray Level Co-Occurrence Matrix Based on Diagnostic Ultrasound Images

**DOI:** 10.3390/jcm14072268

**Published:** 2025-03-26

**Authors:** Minkyoung Kim, Kyuseok Kim, Hyun-Woo Jeong, Youngjin Lee

**Affiliations:** 1 Department of Health Science, General Graduate School of Gachon University, 191, Hambakmoe-ro, Yeonsu-gu, Incheon 21936, Republic of Korea; 2 Institute of Human Convergence Health Science, Gachon University, 191, Hambakmoe-ro, Yeonsu-gu, Incheon 21936, Republic of Korea; 3 Department of Biomedical Engineering, Eulji University, 553, Sanseong-daero, Sujeong-gu, Seongnam-si 13135, Republic of Korea; 4 Department of Radiological Science, Gachon University, 191, Hambakmoe-ro, Yeonsu-gu, Incheon 21936, Republic of Korea

**Keywords:** diagnostic ultrasound image, gray level co-occurrence matrix, texture analysis, kidney and gallbladder stones, posterior acoustic shadow

## Abstract

**Background/Objectives:** Accurate diagnosis during ultrasound examinations of patients with kidney and gallbladder stones is crucial. Although stone areas typically show posterior acoustic shadowing on ultrasound images, their accurate diagnosis can be challenging if the shaded areas are vague. This study proposes a method to improve the diagnostic accuracy of kidney and gallbladder stones through texture analysis of ultrasound images. **Methods:** Two doctors and three sonographers evaluated abdominal ultrasound images and categorized kidney and gallbladder stones into groups based on their predicted likelihood of being present: 50–60%, 60–80%, and ≥80%. The texture analysis method for the posterior acoustic shadows generated from ultrasound images of stones was modeled using a gray level co-occurrence matrix (GLCM). Average values and 95% confidence intervals were used to evaluate the method. **Results:** The three prediction classes were clearly distinguished when *GLCM_Contrast_* was applied to the ultrasound images of patients with kidney and gallbladder stones. However, *GLCM_Correlation_*, *GLCM_Energy_*, and *GLCM_Homogeneity_* were found to be difficult for analyzing the texture of shadowed areas in ultrasound images because they did not clearly or completely distinguish between the three classes. **Conclusions:** Accurate diagnosis of kidney and gallbladder stones may be possible using the GLCM texture analysis method applied to ultrasound images.

## 1. Introduction

Ultrasound is a diagnostic technology that utilizes high-frequency sound waves, which are inaudible to the human ear, to acquire images by reflecting and transmitting these waves through the human body. This technology is widely used for detecting lesions. Among the different clinical ultrasound examinations, abdominal scanning is designated as a representative method for health checkups in many countries [1]. Abdominal ultrasound is significantly advantageous as it enables the noninvasive and nondestructive observation of lesions in abdominal organs [2,3].

Kidney and gallbladder stones are common clinical conditions detected through ultrasound imaging. Kidney stones have a high recurrence rate of up to 50% within 5–10 years, while gallbladder stone prevalence increases with risk factors such as obesity, high-fat diets, metabolic disorders, and aging (over 20% in individuals above 60) [4]. Geographical and environmental factors influence prevalence, with kidney stones more common in hot, arid climates due to dehydration and gallbladder stones more frequent in populations consuming high-cholesterol diets.

When assessing calculus using ultrasound, certain patterns are consistently observed, helping to distinguish stones from other conditions. Kidney stones typically appear as hyperechoic (bright) foci with posterior acoustic shadowing due to the high reflectivity of the stone material [5]. Small stones (≤5 mm) may not always produce a clear shadow, but they can be detected using Doppler ultrasound, which may reveal a twinkle artifact, an aliasing color signal behind the stone. Stones located in the renal pelvis or ureter may cause obstruction, leading to hydronephrosis, which can be identified as an anechoic fluid-filled area within the collecting system [6]. For gallbladder stones, the typical ultrasound pattern includes mobile hyperechoic stones with posterior acoustic shadowing. When the patient’s position changes, gallstones tend to move, distinguishing them from polyps, which remain stationary. In cases of microlithiasis, a low-level internal echo is observed without significant shadowing [7].

However, when doctors and sonographers in the radiology department diagnose stones using ultrasound images, the accuracy decreases if the shadowed areas are not clearly visible. In situations where it is difficult to visually analyze stones on ultrasound images, a new method for analyzing the texture of the image can be highly effective.

Analyzing features on ultrasound images involve extracting and interpreting various aspects of the images to assist in diagnostic or therapeutic applications. Because speckle noise is inherent in ultrasound, significant efforts have been made to develop filtering and adaptive noise reduction methods to enhance image clarity. Non-local means filtering, anisotropic diffusion, and deep learning-based noise reduction models are commonly researched techniques to improve feature visibility [8,9,10]. Although removing speckle noise reduces image distortion and aids in stone identification, there is a limit to the amount of calcification that can be visually detected as a stone. Accurate boundary detection and segmentation are crucial for quantifying structures, such as tumor or organ boundaries, using ultrasound.

Techniques such as active contours, watershed segmentation, and convolutional neural networks have proven instrumental in automatically segmenting complex structures within noisy ultrasound images [11,12,13]. This method is advantageous as it can assist in diagnosing stones after extracting the stone region. However, it also has limitations, as it requires accurate definition and labeled data for stone morphology. Characterizing the substance of interest can also be an effective approach for determining whether it is a stone. Quantitative features, such as texture, shape, and contrast, are extracted to detect and classify diseases. For example, in breast ultrasound, features such as lesion shape, margin, and echotexture are analyzed to distinguish between benign and malignant tissues [14,15], often leveraging machine learning classifiers, including support vector machines, for automated diagnosis [16].

Discrete wavelet transform (DWT) decomposes an image into components at various scales using filters designed to capture subtle intensity variations. This decomposition facilitates the extraction of detailed textural features that are significant for distinguishing malignant lesions from benign lesions [17]. However, DWT involves the multiscale decomposition of images, which requires considerable processing power and time, particularly at higher levels of decomposition. For real-time applications, such as ultrasound imaging, which involves continuous frame-by-frame analysis, this complexity can result in delays, making real-time processing challenging. In addition, the standard DWT does not provide information about directional textures, which is crucial for differentiating between tissue structures in ultrasound images. This limitation can compromise the clarity and detail required for an accurate real-time interpretation. Techniques such as the dual-tree complex wavelet transform are often preferred for addressing this issue; however, they require further complexity.

A gray level co-occurrence matrix (GLCM) [18] can effectively extract features and independently address limitations associated with previous methods. The GLCM can calculate various textural features that describe the spatial distribution and pattern of pixel intensities, aiding in distinguishing between different types of tissues or textures. The commonly used features include contrast, correlation, energy, and homogeneity. The GLCM calculations are sensitive to the direction and distance parameters between pixels, which are typically represented at angles (0°, 45°, 90°, and 135°) [19]. Changing these parameters enables texture analysis across various orientations and scales that can be tuned for specific image analysis requirements.

This study aimed to investigate GLCM features in the shadowed regions of ultrasound images and assess their correlation with stones for the accurate detection and diagnosis of stones. Four GLCM features were calculated for the shadowed areas according to the degree of calcification, and their significance was determined using statistical analysis. The proposed method was implemented, and clinical experiments were conducted to demonstrate its usefulness.

## 2. Materials and Methods

### 2.1. Ultrasound Image Acquisition

This retrospective study was approved by the Institutional Review Board (IRB) of Gachon University (1044396-202411-HR-185-01), which waived the need for informed consent owing to its retrospective design. The IRB review waiver was approved on 20 November 2024.

A LOGIQ S7 R3 Expert (GE Healthcare, Chicago, IL, USA) diagnostic ultrasound device and a convex probe were used for the ultrasound examination of the kidneys and gallbladder. From 2022 to 2023, 52 images of kidney stones and 66 images of gallbladder stones were acquired using abdominal ultrasound. All ultrasound images were independently reviewed and verified by two doctors and three sonographers. The images were then categorized into three stone diagnostic probability groups: 50–60%, 60–80%, and ≥80%, based on the degree of shadowing behind the substance. Table 1 summarizes the classification of ultrasound images of kidney and gallbladder stones.

### 2.2. Proposed Framework to Determine Kidney and Gallbladder Stones Using the GLCM

**Figure 1:**
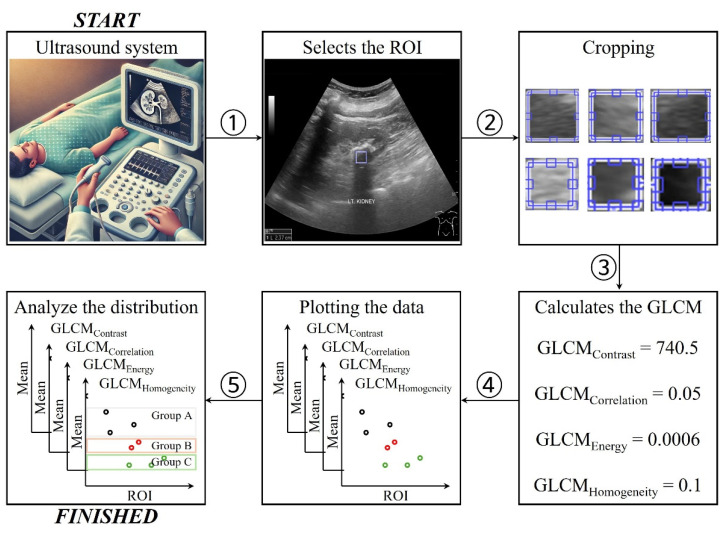
Simplified framework for analyzing the distribution of kidney and gallbladder stones according to the degree of calcification using gray level co-occurrence matrix (GLCM) functions.

Figure 1 shows a simplified scheme for determining kidney and gallbladder stones in the acoustic shadow area using GLCM features. Briefly, images of the kidney and gallbladder were acquired using a diagnostic ultrasound imaging modality. The region of interest (ROI) was set to the shadow that occurs behind the area determined to be a stone in the acquired ultrasound image (Figure 1 ①). The size and location of the ROI were chosen by the users based on their experience. The selected ROIs were cropped to create a single patch, and the process was repeated on the images of patients with known or suspected kidney and gallbladder stones (Figure 1 ②). The GLCM function was calculated based on the obtained patches.

The GLCM properties used were ‘contrast’, ‘correlation’, ‘energy’, and ‘homogeneity’, which were expressed as follows (Figure 1 ③):

(1) $${GLCM}_{Contrast}=\sum_{i,j}{\left|i-j\right|}^{2}p\left(i,j\right),$$

(2) $${GLCM}_{Correlation}=\sum_{i,j}\frac{\left(i-\mu i)(j-\mu j)p(i,j\right)}{\sigma_{i}\sigma_{j}},$$

(3) $${GLCM}_{Energy}=\sum_{i,j}{p(i,j)}^{2},$$

(4) $${GLCM}_{Homogeneity}=\sum_{i,j}\frac{p(i,j)}{1+\left|i-j\right|},$$

where p(i,j) is the probability value in the GLCM for the occurrence of intensity values i and j at a given offset; μi and μj are mean values of the row and column indices across the GLCM; and $\sigma_{i}$ and $\sigma_{j}$ are standard deviations of the row and column indices in the GLCM. The GLCM contrast formula measures the intensity contrast between a pixel and its neighbors across the entire image. This provides a sense of the extent to which local variations in intensity (sharp changes in intensity) exist in an image. Lower contrast values imply more uniform or smoother textures, where the intensity differences between the pixels are minimal. The GLCM correlation function calculates the degree of linear dependency between the pixel intensities in a particular spatial relationship. This feature indicates how correlated a pixel is with its neighbor over the entire image, with higher values suggesting stronger relationships between the pixel intensities. A value of 1 or −1 indicates a positive or negative linear relationship between intensities. A value of 0 indicates no correlation. The GLCM energy metric measures the uniformity or “orderliness” of pixel intensity patterns in an image. It reflects the sum of the squared elements in the GLCM and is particularly useful for identifying the texture consistency within an image. Higher energy values indicate a more uniform or homogeneous texture in the image because larger values in the GLCM dominate the sum when squared. In the GLCM, homogeneity is a measure of the similarity of pixel intensities, indicating the similarity of pixels in close proximity. A high homogeneity value indicates that the texture is uniform or smooth, with similar intensities between adjacent pixels.

GLCM values calculated from the acquired patches were plotted (Figure 1 ④). Subsequently, the mean and 95% confidence interval (CI) were calculated according to the degree of calcification of kidney and gallbladder stones to check whether the groups were formed (Figure 1 ⑤). The proposed framework was implemented on a standard workstation (OS: Window 10 with a CPU clocked at 2.13 GHz, and equipped with 64 GB RAM). The GLCM functions used the built-in MATLAB function *graycoprops* (R2022a, MathWorks Corp., Natick, MA, USA). The proposed method took an average of less than one minute from computation to results when performed by two doctors and three sonographers under the computer conditions described. The proposed method can be applied without any changes to the existing system.

### 2.3. Evaluation Method of Ultrasound Image Texture

To evaluate whether the four GLCM characteristics reflected the shadowing of stones, the ROI was set in the area where shadowing occurred. The average values and 95% CIs obtained from ROIs were calculated.

## 3. Results

**Figure 2:**
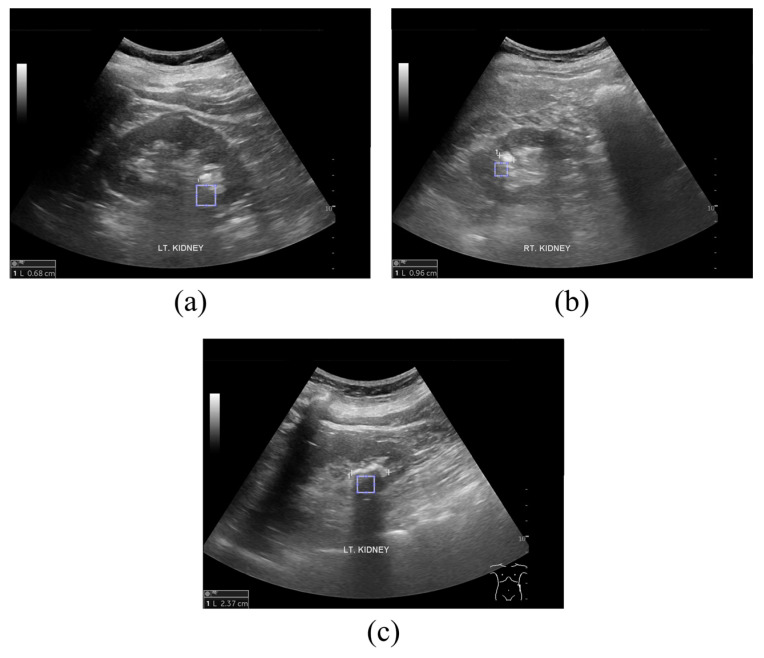
Kidney stone ultrasound images acquired for GLCM analysis. Sample ultrasound images of suspected kidney stones, with probabilities of (**a**) 50–60%, (**b**) 60–80%, and (**c**) ≥80%, analyzed by doctors and sonographers.

Figure 2 shows sample ultrasound images of kidney stones according to the prediction probability of the diagnosis. Figure 2a–c display sample ultrasound images where kidney stones are predicted with a probability of 50–60%, 60–80%, and ≥80%, respectively. For the texture analysis of the ROIs using GLCM, the shadowed areas were appropriately set behind the regions where kidney stones were expected to be located.

**Figure 3:**
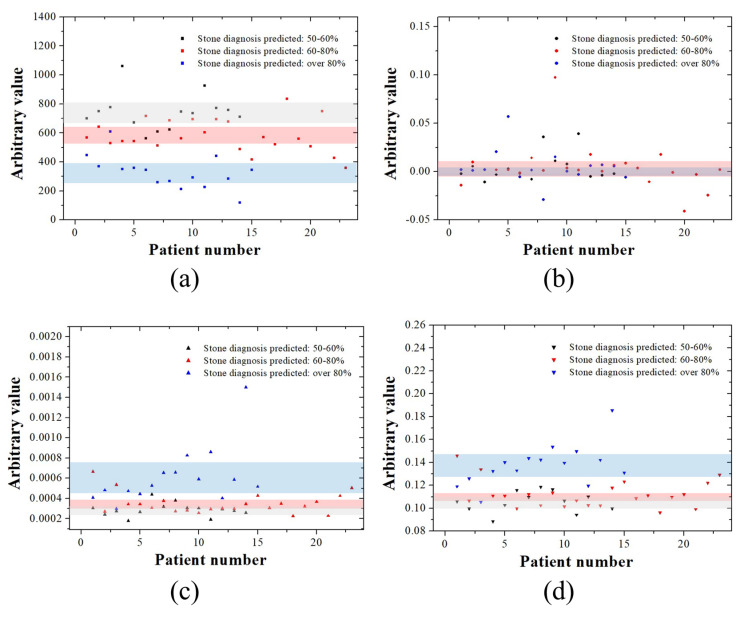
Analysis of GLCM characteristics with respect to the expected diagnosis rate of kidney stones. (**a**) ${GLCM}_{Contrast}$, (**b**) ${GLCM}_{Correlation}$, (**c**) ${GLCM}_{Energy}$, and (**d**) ${GLCM}_{Homogeneity}$ analysis graphs for the posterior acoustic shadow region of kidney stones. Black, red, and blue shades in the graph represent the range of confidence interval values for ultrasound images with suspected kidney stones, categorized into probabilities of 50–60%, 60–80%, and ≥80%, respectively.

Figure 3 shows the arbitrary values for each kidney stone ultrasound image according to the four characteristics of the GLCM. The mean ${GLCM}_{Contrast}$ values were calculated to be 743.31 [95% CI, 676.68–809.94], 582.93 [95% CI, 535.89–629.97], and 328.70 [95% CI, 269.90–387.51] for kidney stone ultrasound images with a 50–60%, 60–80%, and ≥80% probability, respectively. The mean ${GLCM}_{Correlation}$ values were 0.0151 [95% CI, −0.0032–0.0126], 0.0041 [95% CI, −0.0057–0.0140], and 0.0050 [95% CI, −0.0042–0.0008] for kidney stone ultrasound images with a 50–60%, 60–80%, and ≥80% probability, respectively. The mean ${GLCM}_{Energy}$ values were 0.000288 [95% CI, 0.000253–0.000324], 0.000351 [95% CI, 0.000308–0.000393], and 0.000615 [95% CI, 0.000470–0.000761] for kidney stone ultrasound images with a 50–60%, 60–80%, and ≥80% probability, respectively. The mean ${GLCM}_{Homogeneity}$ values were 0.1054 [95% CI, 0.1010–0.1099], 0.1121 [95% CI, 0.1072–0.1171], and 0.1376 [95% CI, 0.1283–0.1468] for kidney stone ultrasound images with a 50–60%, 60–80%, and ≥80% probability, respectively. By analyzing the CI area in black (50–60%), red (60–80%), and blue (≥80%), we confirmed that ${GLCM}_{Contrast}$ was clearly divided into three types (Figure 3a). ${GLCM}_{Correlation}$ showed almost no distinction between the three color shades based on the CI values (Figure 3b). Some boundaries and separation areas were observed in ${GLCM}_{Energy}$ and ${GLCM}_{Homogeneity}$ (Figure 3c,d).

**Figure 4:**
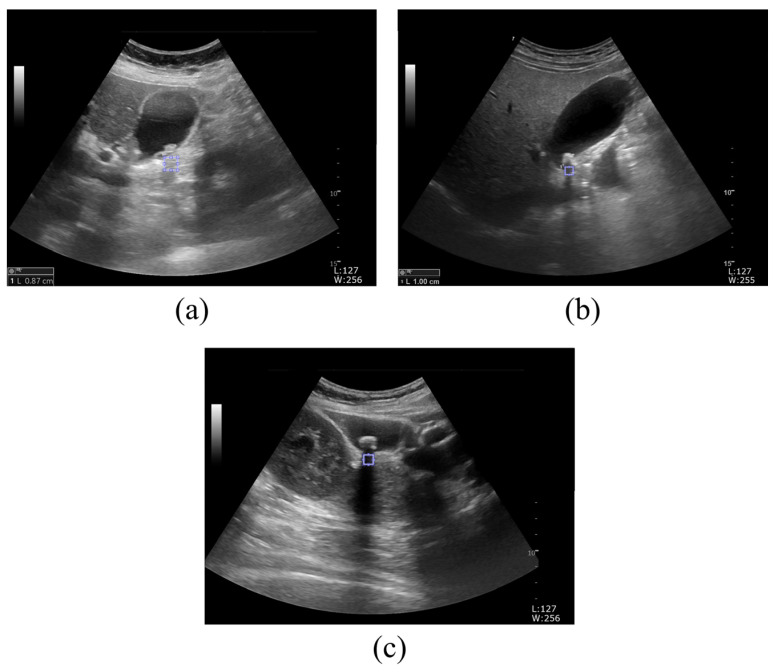
Gallbladder stone ultrasound images acquired for GLCM analysis. Sample ultrasound images of suspected gallbladder stones, with probabilities of (**a**) 50–60%, (**b**) 60–80%, and (**c**) ≥80%, analyzed by doctors and sonographers.

Figure 4 depicts sample ultrasound images of gallbladder stones according to the prediction probability of the diagnosis. Figure 4a–c display sample ultrasound images where gallbladder stones are predicted with a 50–60%, 60–80%, and ≥80% probability, respectively. For the texture analysis of ROIs using GLCM, the shadowed areas were appropriately set behind the regions where gallbladder stones were expected to be located.

**Figure 5:**
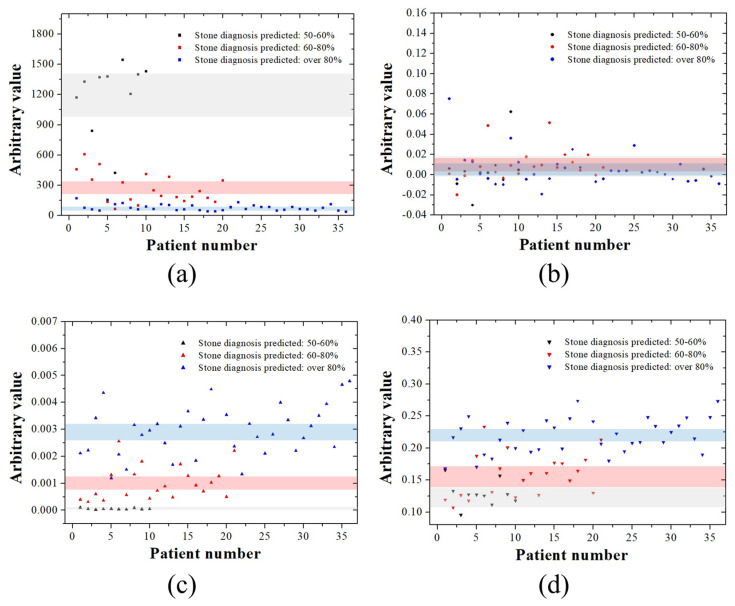
Analysis of GLCM characteristics with respect to the expected diagnosis rate of gallbladder stones. (**a**) ${GLCM}_{Contrast}$, (**b**) ${GLCM}_{Correlation}$, (**c**) ${GLCM}_{Energy}$, and (**d**) ${GLCM}_{Homogeneity}$ analysis graphs of the posterior acoustic shadow region of gallbladder stones. Black, red, and blue shades in the graph represent the range of confidence interval values for ultrasound images with suspected gallbladder stones, categorized into probabilities of 50–60%, 60–80%, and ≥80%, respectively.

Figure 5 shows the arbitrary values for each gallbladder stone image according to the four characteristics of the GLCM. The mean ${GLCM}_{Contrast}$ values were calculated to be 1208.24 [95% CI, 999.17–1417.32], 258.93 [95% CI, 194.45–323.41], and 79.74 [95% CI, 69.09–90.38] for gallbladder stone ultrasound images with a 50–60%, 60–80%, and ≥80% probability, respectively. The mean ${GLCM}_{Correlation}$ values were 0.0044 [95% CI, −0.0010–0.0188], 0.0102 [95% CI, 0.0034–0.0170], and 0.0048 [95% CI, −0.0007–0.0103] for gallbladder stone ultrasound images with a 50–60%, 60–80%, and ≥80% probability, respectively. The mean ${GLCM}_{Energy}$ values were 0.000054 [95% CI, 0.000040–0.000068], 0.001020 [95% CI, 0.000750–0.001290], and 0.002925 [95% CI, −0.002616–0.003233] for gallbladder stone ultrasound images with a 50–60%, 60–80%, and ≥80% probability, respectively. The mean ${GLCM}_{Homogeneity}$ values were 0.1288 [95% CI, 0.1162–0.1413], 0.1573 [95% CI, 0.1427–0.1719], and 0.2188 [95% CI, 0.2098–0.2277] for gallbladder stone ultrasound images with a 50–60%, 60–80%, and ≥80% probability, respectively. By analyzing the CI areas marked in black (50–60%), red (60–80%), and blue (>80%), we confirmed that ${GLCM}_{Contrast}$ and ${GLCM}_{Energy}$ clearly differentiated the three categories (Figure 5a,c). However, ${GLCM}_{Correlation}$ showed almost no distinction between the three color shades based on the CI values (Figure 5b). ${GLCM}_{Homogeneity}$ was divided into three types with subtle differences in spacing (Figure 5d).

## 4. Discussion

This study demonstrated that the contrast feature of the GLCM exhibited the strongest linear relationship with the shadowing observed behind suspected stones. Regardless of the type of stone in the kidney or gallbladder, the texture of the posterior acoustic shadow could be analyzed using the ${GLCM}_{Contrast}$. In addition to the high linear contrast, the energy and homogeneity feature may also contribute to analyzing the texture of the shaded area, depending on the situation. However, the correlation-based GLCM values in the ultrasound images of both diseases showed limited linearity. Therefore, for more accurate diagnosis of stones using GLCM in ultrasound images, it is recommended to use contrast-based features for texture analysis.

When the diagnosis probability was divided into probabilities of 50–60%, 60–80%, and ≥80% according to the strength of the posterior acoustic shadow of kidney stones and gallbladder stones, the 95% CI values tended to be clearly lower in ${GLCM}_{Contrast}$ and slightly higher in ${GLCM}_{Energy}$ and ${GLCM}_{Homogeneity}$ in the order of probabilities of 50–60%, 60–80%, and ≥80%. This means that the image in the ROI area becomes increasingly uniform and smooth. On the other hand, the 95% CI value of correlation remained almost similar regardless of the degree of shadowing. Correlation characteristics depend on the linear relationship between pixels, so they may show changes that differ from trends in contrast, homogeneity, and energy. Even if the image becomes uniform, if the distribution pattern of pixel intensity values does not change significantly, the values remain similar. For example, if the texture maintains a certain pattern, such as a grid shape, this means that directionality is maintained. When the image is homogenized, the difference in intensity values is reduced and overall simplified, but if the arrangement direction of the pattern does not change, it does not change significantly. In this study, the texture of the posterior region tends to become more uniform and softer as the degree of shading becomes stronger, but there is no significant change in the anatomical structure behind the kidney or gallbladder stones, so it is possible to consider the possibility that the pixel intensity pattern may remain similar.

As the accuracy of diagnosing kidney and gallbladder stones using ultrasound improves with the analysis of contrast characteristics using GLCM, additional examinations can help guide appropriate treatment directions. For kidney stones, even in the absence of pain when a stone is discovered early, future management and prevention can be expected through periodic observation and methods such as increasing fluid intake, diuresis, or dietary management [20,21]. Patients with painful stones can actively undergo treatment through extracorporeal shock wave lithotripsy [22,23]. For the gallbladder, distinguishing stones from polyps can be challenging if the shadowing behind the stones is unclear and the movements are not well-defined, even with changes in the patient’s position. We anticipate that using GLCM-based analysis will aid in the early differentiation of polyps and stones in ultrasound images [24]. Since 5–10% of gallbladder polyps have the potential to become cancerous, continuous follow-up for size and shape is necessary [25]. If gallbladder stones are diagnosed, follow-up observation, oral bile acid dissolution therapy, and cholecystectomy may be considered, depending on the severity of symptoms, size of the lesion, and presence or absence of polyps [26].

The proposed method offers a quantitative indicator for kidney and gallbladder stones, making it sufficiently applicable in clinical settings. However, certain aspects still need to be considered as follows:

(1) As a retrospective study, it is subject to inherent biases, including selection bias and variability in imaging protocols.
(2) There may be potential variability in image quality and operator-dependent factors inherent to ultrasound imaging.
(3) The feature analysis was performed using only GLCM, and further research based on various texture analysis matrices is needed.

First, the retrospective nature also limits our ability to control for potential confounding factors, such as differences in ultrasound acquisition settings and patient characteristics. Additionally, the study was conducted in a single-center setting, which may limit the generalizability of the results to broader populations. Future prospective studies with standardized imaging protocols and a larger, more diverse cohort are necessary to validate our findings and assess their clinical applicability. Second, the location of the ROI for the acoustic shadow behind the stone must be carefully selected. In the proposed algorithm, the GLCM is calculated using the extracted patches after setting the ROI. When computing the GLCM, changing the ROI size can result in the extraction of different feature values, affecting the consistency of the results. In research, it is important to evaluate the reproducibility of ultrasound features at different ROI sizes to identify features that are more reproducible at certain sizes. For example, if the ROI is too small, it may not reflect sufficient information from the area. Conversely, if it is too large, it may include unnecessary information from the surrounding area, potentially reducing the accuracy of the analysis [27]. Therefore, selecting the correct size of the ROI is critical for improving the quality of the analysis. Further research into how changes in ROI size affect the results will enhance the reliability of these models. Studies evaluating the impact of ROI size on the diagnostic performance, particularly in shear wave elastography, have identified that diagnostic performance differs when differentiating between benign and malignant solid breast lesions using small versus large ROIs. In this context, a small ROI, located in the hardest part of the lesion, provided better diagnostic performance than a large ROI that encompassed the entire lesion. Significant differences were observed in maximum elasticity, mean elasticity, minimum elasticity, and standard deviation values. Therefore, further research on ROI size using our proposed method is planned [28]. In addition, the zone setup was based on the experience of a diagnostic medical sonographer. Their experience significantly influenced the accuracy of kidney and gallbladder stones diagnoses [29]. A review highlighted that sonography remains the initial imaging technique for evaluating patients with suspected acute calculous cholecystitis due to its high sensitivity for detecting gallbladder stones [30]. This review also emphasized that the accuracy of ultrasonography is closely linked to the operator’s experience and expertise. These studies underscore the critical role of the sonographer’s experience in accurately diagnosing kidney and gallbladder stones, thereby influencing patient outcomes and treatment decisions. Although this study involved experienced sonographers in extracting and classifying kidney and gallbladder stone images, individual human error and varying levels of experience may have influenced the results. To overcome this limitation, it is necessary to analyze the results derived from multiple sonographers using receiver operating characteristic curves, which we are preparing to conduct as part of a follow-up study. Finally, further research using various texture analysis matrices is required. The proposed method is a quantitative classification study based on the GLCM, a basic texture analysis matrix that uses the features of the acoustic shadow behind the stone. Recently, various texture analysis methods, such as Gaussian Markov random fields (GMRFs) [31] and the gray level run length matrix (GLRLM) [32], have been introduced in addition to the basic GLCM, and their usefulness has been confirmed. GMRF-based methods model the spatial dependence between pixel intensities to characterize textures, providing a probabilistic framework for texture representation. Studies have shown that combining GMRFs with statistical methods, such as the GLCM, improves the classification accuracy in texture analysis. The GLRLM evaluates texture by assessing the length of consecutive pixels with the same intensity and capturing patterns related to texture roughness and directionality. Studies have demonstrated the effectiveness of applying GLRLM features to histopathological image classification to distinguish between normal and malignant samples. The application of these functions to the proposed method may yield more meaningful correlations. Considering these points, we expect that the proposed method will be useful for extracting and discriminating kidney and gallbladder stones.

## 5. Conclusions

In this study, we demonstrate that GLCM texture analysis applied to ultrasound images holds potential for improving the diagnostic accuracy of kidney and gallbladder stones. This study revealed that the contrast feature exhibited the most pronounced linearity. In contrast, the correlation feature showed no linearity at all. Additionally, the energy and homogeneity features did not exhibit linearity as distinctly as the contrast feature. These findings suggest that the proposed method can be used as a key indicator for the identification or diagnosis of kidney and gallbladder stones. While our findings suggest that this method may enhance stone detection, further clinical validation is necessary to establish its reliability in diverse patient populations. The integration of texture analysis into routine ultrasound assessments could support radiologists and clinicians in distinguishing pathological conditions more effectively. Future studies should focus on refining texture-based diagnostic algorithms and evaluating their impact on clinical decision-making to maximize their utility in real-world practice.

## Figures and Tables

**Table 1:** Detailed number of images based on the probability of suspected kidney and gallbladder stones.

Disease Type	Expected Probability of Diagnosis		
	Number of Images with 50–60% Probability	Number of Images with 60–80% Probability	Number of Images with ≥80% Probability
Kidney stone	14	23	15
Gallbladder stone	10	21	35

## Data Availability

The raw data supporting the conclusions of this article will be made available by the authors on request.
